# Microbiological Hygiene Quality of Thermal Muds: A Pilot Study in Pelotherapy Facilities of the Euganean Thermal District (NE Italy)

**DOI:** 10.3390/ijerph17145040

**Published:** 2020-07-13

**Authors:** Tatjana Baldovin, Irene Amoruso, Fabrizio Caldara, Alessandra Buja, Vincenzo Baldo, Silvia Cocchio, Chiara Bertoncello

**Affiliations:** 1 Department of Cardiac, Thoracic, Vascular Sciences and Public Health, Unit of Hygiene and Public Health, University of Padua, Via L. Loredan, 18-35131 Padua, Italy; tatjana.baldovin@unipd.it (T.B.); alessandra.buja@unipd.it (A.B.); vincenzo.baldo@unipd.it (V.B.); silvia.cocchio@unipd.it (S.C.); chiara.bertoncello@unipd.it (C.B.); 2 Pietro d’Abano Center for Thermal Studies, Via Jappelli, 5-35031 Abano Terme (PD), Italy; fabrizio.caldara@centrostuditermali.org

**Keywords:** thermal mud, pelotherapy, peloids, SPA, microbiological quality

## Abstract

Evaluation of hygienic aspects of thermal mud microbiology is still neglected. This study evaluates the microbiological hygiene quality of thermal muds, providing a comprehensive assessment of the whole mud cultivation chain. Maturing mud, peloid and used mud samples were collected twice in a year from 30 SPAs of the Euganean Thermal District, NE Italy. Samples were processed with an ad hoc laboratory method. The following indicator parameters were assessed: Total Count at 22, 37 and 55 °C; total coliforms; *Escherichia coli*; enterococci; *Staphylococcus aureus*; *Pseudomonas aeruginosa*; sulfite-reducing clostridia; dermatophytes. Statistical significance of differences between the two sampling campaigns and correlation between temperature and indicator parameters were evaluated. One-hundred eighty samples were analyzed. Widespread presence of environmental species was found, as well as hints of possible microorganism transfer from the patient’s skin to the mud. Proper setting of thermal water temperature resulted critical, in terms of hygienic quality. Although optimal maturation should be granted (thermal water at 30–42 °C), a pasteurization step at 60–65 °C is strongly recommended to sanitize peloids before pelotherapy. Facilities re-using thermal muds should also implement a regeneration step at ≥65 °C. Core evaluation of thermal mud hygienic quality could encompass the following guidelines: absence (i.e., 0 colony forming units (CFU)/g) of *E. coli*, *P. aeruginosa*, *S. aureus* and dermatophytes.

## 1. Introduction

Beneficial properties of clay minerals and especially of thermal muds (TMs) are well known and established: their uses in both historical and modern times are thoroughly discussed in a dedicated review [1]. Among the many curative applications, the ancient practice of pelotherapy has been carried out for centuries worldwide [2,3] and it gained popularity also for wellness purposes [4]. Pelotherapy consists in the application of hot TMs (40 ± 2 °C) in a thick-layer, directly on the skin of the patient that is then covered with an insulating cloth, in order to preserve heat.

Pelotherapy has a stimulatory, antiphlogistic, analgesic action [1,4,5] and it is recommended either as treatment or adjuvant therapy for rheumatic disorders and other musculoskeletal conditions, e.g., osteoarthritis [6,7,8], fibromyalgia [9,10], rheumathoid arthritis [9], low back pain [11] and traumatic injuries [12]. Pelotherapy is suggested also for other conditions, e.g., dermatological disorders [13], neuro- and vasculopathies [14,15] and to improve stress resilience [16].

The overall quality of TMs is determined by several factors, i.e., composition of raw mud, thermal water characteristics and maturation procedure. In fact, TMs gain their therapeutic properties during the maturation process, in which mud is blended with thermal water under specific conditions [12,17]. Complex inorganic and organic changes of the mud matrix lead to the improvement of physico-chemical, rheological and biological properties required for an effective pelotherapy [1,2,12]. Most pelotherapy centers employ raw muds occurring in situ, but where exploitation of natural reserves is strictly regulated [18], TMs are currently regenerated, i.e., after the first application on the patient they are matured and used anew [4]. In the past, some authors discussed the need to establish standard quality criteria for TMs intended to be used in therapy [3,4]. Several studies evaluated chemical, mineralogical, radiological and granulometric properties of raw muds under the perspective of human health safety e.g., [19,20,21,22]. The mobility of hazardous chemical elements possibly contained in clay materials was also assessed with peculiar in vitro leaching tests [23,24].

So far, biological investigations mainly addressed the characterization of thermophilic microorganisms (e.g., diatoms and cyanobacteria) involved in the maturation process and release of therapeutically active biogenic compounds [2,3,25,26,27]. On the contrary, hygienic aspects of TMs microbiology have been contemplated only by a few studies [3,28,29,30,31,32]. However, no exhaustive research on microbiological hygiene quality of TMs has been published so far. Studies evaluating either the effectiveness of mud cultivation in terms of sanitization or the possible transfer of microorganisms from patient to TM are also missing.

The present work aims at investigating the microbiological quality of TMs from the microbiological hygiene perspective. A dedicated laboratory method was implemented and a set of suitable indicator parameters was tested. Especially, microbial safety of every step of the TM cultivation process was evaluated to highlight critical points and peculiar contamination risks.

## 2. Materials and Methods

### 2.1. Sampling Sites

TM samples were collected from 30 facilities of the Euganean thermal basin (NE Italy), each performing pelotherapy either as private spa or in convention with the Italian National Health System. Each facility was surveyed twice in a year, respectively during the two thermal tourism high seasons, i.e., spring sampling (SS) and autumn sampling (AS).

### 2.2. Sample Collection

From each cultivation plant site, 6 different mud samples were collected. In detail, three different kinds of muds were sampled during both SS and AS: maturing mud (M); peloids (P) and used mud undergoing regeneration (R). A steel core-drilling device with a diameter of 6 cm was used to collect mud samples. An inner piston collects a mud cylinder of 20 cm in height, thus sampling the mud layer involved by maturation biological processes [33]. The core-drilling device was rinsed with water and thoroughly flamed with a field Bunsen burner before the collection of a different sample. Each mud sample was extruded from the core-drilling device into a sterile Stomacher^®^ bag (Seward GmbH, Worthing, UK). Samples that could not be collected with the core-drilling device (i.e., automated dispensers) were directly poured inside Stomacher^®^ bags. Samples were carried to the laboratory by cooled transport (4 °C) and processed within 10 h from collection. Temperature was also recorded for each sampling point. 

### 2.3. Microbiological Methods

Prior to analysis, the content of each Stomacher^®^ bag was manually kneaded for 30 s to roughly uniform the sample. A 1:10 dilution was prepared for each sample by suspending 100 ± 3 g of mud in 900 mL of Dulbecco Phosphate Buffer Saline, inside a sterile 2 L Erlenmeyer flask. The flask was then placed on an orbital shaker at 300 rpm for 10 min or until complete suspension was achieved. The flask was further maintained under slow agitation during laboratory testing procedures, in order to avoid sedimentation of the sample and, possibly, lower bacteria recovery.

Selected indicator parameters were: total viable count (TVC), total coliforms, *E. coli*, enterococci, *S. aureus, P. aeruginosa*, and sulfite-reducing clostridia and dermatophytes fungi. Table 1 reports the full list of indicator parameters, growth media (Biolife Italiana, Milano, Italy) and incubation conditions. TVC was evaluated by pour plate method at 22, 37 and 55 °C by seeding 1 mL of sample. For each sample, multiple dilutions were prepared and tested (i.e., 1:10, 1:100 and 1:1000), in order to obtain results within the countable range of 30–300 colony forming units (CFU) per plate. The other parameters were evaluated by membrane filtration technique. Three different volumes (i.e., 1, 5 and 10 mL) of the 1:10 diluted sample were filtered on 0.45 µm sterile cellulose acetate membranes (Sartorius-Stedim Biotech, Goettingen, Germany), to grant readability of the plate (i.e., 20–200 CFU per plate). Membranes were then transferred on Ø 60 mm petri dishes containing dedicated agar media.

### 2.4. Statistical Testing

Statistical analysis was conducted with software SOFA Statistics v1.5.2 (Paton-Simpson & Ass. Ltd, Wellingotn, New Zealand). Non-parametric tests were applied. Wilcoxon Signed Rank Test was used to assess differences between SS and AS paired data. Correlations between temperature and TVCs were evaluated with Spearman’s R test. The Mann–Whitney U test was run to assess interaction of temperature with indicator parameters other than TVCs, in terms of presence/absence.

## 3. Results

Relevant characteristics of the 30 facilities considered in the study are described in Table 2. Traditional mud cultivation (T) in 4 × 4 × 1 m concrete tanks was implemented by 26 facilities; 2 facilities employed a mechanized plant with 20 m^3^ cylindrical iron silos (S) and 2 had a hybrid system (H) with maturation in concrete tanks and storage of TMs in silos. Moreover, distribution of TMs from cultivation area to therapy chambers was also performed with different techniques among different facilities. Direct collection (D) of TMs from maturation tanks and transport with buckets was adopted by 11 sites. The *bagnomaria* hot water bath technique (B) was used by 15 facilities: buckets were filled with TMs and submerged with thermal water in a dedicated tub for at least 24 h prior to therapy. A qualified operator carried TM buckets to the therapy chambers when required. The remaining four facilities were furnished with an automated dispenser (A) that piped TMs from the maturation plant to the therapy chamber. Temperatures of each sampling site are also summarized in Table 2.

### 3.1. Microbiological Analysis

On the whole, 180 TM samples were processed. Total viable counts of SS and AS for all mud typologies and incubation temperatures are reported in Table 3.

Counts for total coliforms and *E. coli* are reported in Table 4. Total coliforms were isolated from 56 samples (31.1%). In detail, total coliforms were found in 21 M (11.7%), 16 P (8.9%) and 19 R (10.6%) mud samples. *E. coli* was detected in 4 samples (2.8%), of whom 2 M (1.1%) and 2 R (1.1%) samples respectively.

Counts for enterococci are reported in Table 5. Enterococci were found in 132 (73.3%) samples, with the same frequency (24.4%) among M, P and R samples.

Among assessed parameters, anaerobic sulfite-reducing clostridia resulted the most abundant group (Table 6): growth was observed in 166 (92.2%) samples, with CFU counts ranging from 3 to 2070 CFU/g.

Table 7 jointly reports the few samples in which *P. aeruginosa* or dermatophytes were retrieved. *P. aeruginosa* was found in 2 (1.1%) AS samples, whereas dermatophytes in 5 SS samples of R mud. *S. aureus* was not found in any of the processed samples.

### 3.2. Statistical Analysis

Comparison of thermal water temperatures recorded during SS and AS returns a significant difference for R tanks only (*p*-value = 0.041; W-statistic = 133), with an average temperature delta of −5 °C during AS. As suggested by raw data, TVCs generally achieve higher counts during AS. Consistently, significant difference between SS and AS is found for 55 °C TVCs of M, P and R mud samples. In addition, the 22 °C TVCs for P and R samples also achieves statistical significance, with higher values during AS (Table 8). 

Significant correlation between TVC and temperature was found only for AS samples. In detail, TVCs at 22, 37 and 55 °C for M as well as at 22 °C and 55 °C for R achieved statistical significance (Table 9). Basically, the higher the temperature the lower the TVC, as hinted by negative ρ coefficients.

The assessment of temperature influence on presence/absence of indicator parameters other than TVCs, returned statistical significance for total coliforms (M, P and R samples), *E. coli* (M samples) and enterococci (R samples) (Table 10). Average thermal water temperature was calculated for presence and absence groups of significant parameters, suggesting that presence always occurred with lower temperatures (Table 11).

## 4. Discussion

The relevance of microbiological hygiene quality has long been neglected for TMs and literature addressing this topic is still quite limited. Sanchez-Espejo et al. [30] evaluated the microbiological compliance of five raw clay samples used to prepare TMs with the limits proposed by the European regulations for medicinal products. Free-living pathogenic amoebas were sought in mud samples by Scaglia et al. [29]. “Disappearance of pathogens” after maturation was reported by Galzigna et al., but no methods nor numeric results were provided [28]. Tentatively, a study from Turkey addressed the microbial contamination of TMs, but methods and results are ambiguous and reproducibility is rather penalized [31]. Quintela et al. [32] published a pilot study on the microbiological quality of maturated volcanic muds: they evaluated the total microbial count at 22 and 37 °C by pour-plate method and total coliforms by membrane filtration technique. In another work they also succinctly discussed, but didn’t assess, microbial content of TMs, suggesting to take into account some indicator parameters, i.e., enterobacteria, *Streptococcus*, *E. coli* and total and fecal coliforms, to ensure safety of patients [3].

Evaluation of microbiological hygiene quality of TMs carried out in the present study encompasses a precise knowledge of both maturation process and pelotherapy protocol, so as to ensure a comprehensive assessment of the whole mud cultivation chain and its critical points. Surveyed facilities are located in the Euganean Thermal District, Veneto Region, NE Italy. This territory is one the oldest thermal areas in Europe and boasts an ancient pelotherapy tradition. In the Euganean thermal basin, virgin mud for pelotherapy is exclusively drawn from two mining concessions, i.e., the Lispida (N 45°16′41″ E 11°46′13″) and the (N 45°16′11″ E 11°44′37″) Arquà Lakes. Currently, a maximum amount of 1000 m^3^ can be extracted per year, as virgin mud is a non-renewable resource [18]. Thermal facilities are thus forced to reuse TM for multiple therapeutic applications. By law, virgin mud must be independently matured by each pelotherapy facility.

Maturation of virgin mud lasts several months, during which the mud is submerged with thermal water. The first hygienic issue concerns the presence of undesirable microorganisms in maturing mud, as the result of a naturally-occurring contamination of the extraction lake sediments. Optimal maturation of TMs in terms of *bioglea* growth and production of therapeutic compounds should be carried out in the 30–42 °C temperature range [33]. Registered temperatures in M sampling sites were compared to the suggested range. A low compliance was generally observed (i.e., about 30% of facilities), since maturing muds were mostly kept at >42 °C. Although affecting the maturation process, this should not penalize hygienic aspects but rather supporting the elimination of undesired environmental microorganisms.

Once maturation is achieved, TMs are traditionally “pasteurized” with thermal water for about 24–48 h prior to therapy [33]. Microbiological analysis of ready-to-use TMs is thus indicative of the efficacy of this intended pre-therapy sanification. Pasteurization of TMs in surveyed facilities was carried out by 75% of facilities at a temperature of ≤60 °C.

As mentioned, due to the locally enforced TM extraction limit [18], all surveyed facilities re-use TMs for multiple treatments. The hygienic concern relevant to this step, regards the possible transfer of microorganisms from the patient’s skin to the applied mud. After treatment, the used mud is immediately transferred in a dedicated collection tank filled with thermal water. The regeneration step lasts about three days. Since regeneration will be followed by a new maturation cycle, correct management of this step, in terms of thermal water temperature, is essential to avoid persistence of microbial contaminants throughout the cultivation process. By the way, only six facilities employed water ≥60 °C amidst SS and AS. Moreover, statistical comparison of thermal water temperatures between the two sampling campaigns suggests that R temperatures were significantly lower during AS (average difference = −5 °C), probably due to the enhanced cooling of thermal water during autumn environmental conditions.

Microbiological analysis of environmental samples usually employs indicator parameters to evaluate the overall quality or potential contamination of samples: contextualization of selected indicator parameters and interpretation of obtained results is hereby presented.

Total viable count is a generic indicator parameter representative for the broad mud microbial colonization, and it is essentially unrelated to pathogen species. TVCs highlighted a copious microbial growth in all samples. Significantly higher TVC counts were observed during AS, if compared to SS. Lower thermal water temperatures reported for R tanks during AS could explain this finding, but still a similar explanation can’t be invoked for M and P samples. Supposedly, environmental variables other than thermal water temperature (e.g., sunlight, air temperature, rainfall) are capable of influencing microbial growth, but an in-depth understanding of such microbial ecological dynamics falls outside the scope of the present research.

Total coliforms encompass a broad class of bacteria commonly found in the natural environment, whereas *E. coli* stands for recent fecal contamination. Total coliforms in M samples should thus not come unexpected. Virgin mud most likely carries coliforms as natural colonizers that further survive in the cultivation plant thanks to the permissive thermal water temperature of maturation tanks. Two M samples (1M-SS; 5M-AS) were found positive also for *E. coli*. Its presence could depend once more on pristine contamination of virgin mud or either on a faulty regeneration procedure of the used mud. Actually, it was impossible to determine whether sampled M mud was virgin mud or regenerated mud. Consistently, temperatures of both M and R sampling sites were quite low for the two facilities (Table 2). Hygienic quality of P samples is of core importance. Whereas presence of total coliforms in P samples can still be tolerated, absence of *E. coli* should be required. Among the analyzed mature mud samples, no one resulted contaminated by *E. coli*. Consistently, presence of *E. coli* in TMs can be prevented by pasteurization with a correct thermal water temperature and contact time. *E. coli* was found in 2 R samples as well (1R-SS with 20 CFU/g; 15R-SS with 24 CFU/g. Thermal water in involved facilities once more was too cold to ensure proper sanification of used mud (Table 2). Similarly to coliforms, the presence of enterococci in natural muds is quite predictable and acceptable. Several studies pointed out how lake sediments are significant reservoirs of enterococci [34]. Members of the *Staphylococcus* genus can be either saprophytic environmental species or microorganisms transferred from the patient’s skin, so that peculiar attention was paid to the possible presence of opportunistic pathogen species *S. aureus* in P and R samples. Nevertheless, no sample resulted positive for this indicator. Among the investigated parameters, anaerobic clostridia, which are commonly found in the environment, represented the most abundant bacterial class. Mud is *per se* an anoxic matrix that favors the proliferation of clostridia and their variable abundance is probably influenced by the mud cultivation and mixing procedures implemented by each facility. *P. aeruginosa* was isolated in two samples (4P-SS and 9M-SS). Consistently, temperature registered for sample 4P and 9M was unsuitable for sanitization purposes (Table 2). Skin-disease causing dermatophyte fungi were sporadically isolated in 5 R samples. Their most plausible origin is direct transfer from patient’s skin to the used mud.

Statistical analysis supports the relevance of thermal water temperature on microbial growth of indicator parameters. Presence or absence of total coliforms resulted significantly linked to different temperature clusters, as shown in Table 11. Similarly, M samples with *E. coli* and R samples with enterococci significantly correlate to lower average temperatures. Although statistical significance was achieved only by the above discussed samples, a similar trend can be observed for all considered indicators and in all mud typologies, confirming the critical role of temperature on the hygienic profiling of TMs.

Overall, some of the selected indicator parameters proved suitable for the assessment of microbiological hygienic quality of TMs. Nevertheless, the need of a precise thermal water temperature guideline value for each mud cultivation phase emerged as a critical issue. A reference protocol addressing both the optimization of cultivation temperatures and setting microbiological quality requirements can now be ventured. During maturation, priority should be given to the correct growth of the bioglea, so that TMs can achieve the best therapeutic quality. A temperature range of 30–42 °C is therefore recommended. Temperatures >42 °C are strongly discouraged since, although enhancing precocious mud sanitization, they can seriously compromise the correct maturation of TMs. Hygienic implications effectively come into play in the pre-therapy pasteurization step. It should be desirable for thermal water in P tanks to be at 60–65 °C. Of future interest, the multidisciplinary evaluation of a temperature range with an upper limit, so as to grant sanification without irretrievably denaturing therapeutic compounds. Furthermore, it should be compulsory to thoroughly sanitize the used mud before they start a new maturation cycle. Thermal water in regeneration tanks should therefore be kept at a temperature of ≥65 °C, for at least 72 h. Disruption of therapeutic compounds and of bio-active microorganisms should not be feared in this case, since the use of a “starter” bioglea can efficiently promote a new maturation process [33]. Of course, since only cultivation techniques of the Euganean basin were considered in the present study, a limitation of the above proposal is that it should be calibrated for other pelotherapy districts. However, the general assessment of hygiene-related critical points of the mud cultivation chain is transferable to other pelotherapy operating facilities.

To the author’s knowledge, there is no national nor international legislation specifically addressing microbial requirements for thermal muds and peloids. Cosmetics regulations can possibly share some similarity, if considering the clayey nature of peloids. The European Regulation (EC) No. 1223/2009 recommends peculiar attention to microbiological purity of topical products to be used on mucous membranes in general, on damaged skin. It also stresses the importance of microbiological quality of products, if used by immunocompromised persons or by elderly people, in reason of their physiological immunosenescence. Safety issues resulting from microbiologically contaminated topical products are sporadic (e.g., infections caused by Gram-negative organisms), yet not negligible [35,36]. Similar recommendations could also suit peloids, especially if taking into account the typical pelotherapy applications and the elderly patients. Nevertheless, hygienic quality of thermal muds should not only focus on raw materials (i.e., virgin mud) and the “finished product” (i.e., the ready-for-therapy peloid). Quality of regenerated muds should definitely be granted by all facilities that reuse them for multiple pelotherapy applications on different patients. Of course thermal muds and peloids need not to be sterile, but they certainly should not be contaminated with undesired or potentially pathogenic microorganisms. 

Among the assessed indicator parameters, some resulted not informative in respect of the hygienic quality characterization of TMs. In detail, TVCs and clostridia resulted too numerically variable among different samples to provide a reliable reference index; total coliforms and enterococci were commonly found as part of the environmental microflora. Recommended indicator parameters and guideline values thus are: absence (i.e., 0 CFU/g) of *E. coli*, *Pseudomonas aeruginosa*, *Staphylococcus aureus* and dermatophytes. Moreover, microbiological hygiene requirements can be reasonably demanded only for ready-for-therapy TMs, after a proper pasteurization step is carried out.

In the end, some considerations about the methodological choices are shared. The present study adopted a classic bacteriology approach after considering some pragmatic and scope-determined aspects. In the first place, a large number of mud samples (i.e., 180) had to be processed, so that the affordability of culture methods certainly played a role. Moreover, culture methods provide precious quantitative details and evaluate the microorganism vitality, a data crucially relevant for a complete risk assessment. Molecular methods such as microarrays or next generation sequencing (e.g., [27,37]) were considered to provide analytical details even beyond the core aim of this pilot research.

## 5. Conclusions

Microbial hygienic quality of TMs was thoroughly assessed after the validation of an ad hoc laboratory method. Analysis of TM samples (i.e., maturing mud, peloids and used mud) highlighted how presence of undesired microorganisms can either result from environmental contamination or transfer from patients’ skin. A core set of suitable indicator parameters for evaluating the microbiological hygiene quality of TMs could encompass *Escherichia coli*, *Pseudomonas aeruginosa*, *Staphylococcus aureus* and dermatophytes. Absence (0 CFU/g) of such indicators is recommended. Proper management of thermal water temperatures throughout the diverse phases of the mud cultivation process represents a critical issue. If, on the one hand, maturation of virgin mud must ensure the achievement of the best therapeutic properties, on the other a pasteurization step of TMs with thermal water ≥ 60 °C should be compulsory just before treatment, so as to grant its hygienic quality as well.

**Glossary of research terms**

In the present work, terms that belong to the field of thermal medicine adhere to the reference glossary proposed by Gomes et al. [2]. Additional voice is given by Authors for *Regeneration*. Concise definitions are hereby reported:

- *Bioglea*: biogenic gelatinous pellicle of yellowish, greenish, grayish or reddish colour, deposited in presence of sulfur-containing waters.
- Maturation: the blending process of muds with thermal water, either in the natural environment or in artificial plants, during which maturing mud achieves therapeutic properties.
- Peloid: maturated mud with therapeutic properties.
- Pelotherapy: external application of thermal muds for therapeutic purposes.
- Regeneration: sanitization process of *used thermal muds.* It is implemented before a new maturation cycle starts by facilities that use muds for multiple pelotherapy applications on different patients.

## Figures and Tables

**Table 1:** Microbiological quality indicator parameters. Agar media and growth conditions, i.e., incubation time and temperature, are hereby reported. PCA—Plate Count Agar; C-EC—Chromogenic Coliform agar; TBX—Tryptone Bile X-GLUC agar; SPS—Sulphite Polymyxin Sulphadiazine; DTM—Dermatophyte agar. * SPS agar plates were incubated in anaerobic conditions, within an anaerobic jar (AnaeroJar 2.5 L, Oxoid Ltd., Basingstoke, UK).

Indicator Parameter	Agar Medium	Incubation Time (hrs)	Growth Temperature (°C)
Total viable count (TVC)	PCA	72	22
		24	37
		24	55
Total coliforms	C-EC	24	37
*Escherichia coli*	TBX	24	44
Enterococci	Slanetz and Bartley	48	37
*Staphylococcus aureus*	Baird-parker	24	37
*Pseudomonas aeruginosa*	Cetrimide	24	37
Sulfite-reducing clostridia	SPS *	24	37
Dermatophytes	DTM	14 days	30

**Table 2:** Characteristics of surveyed facilities and temperature of sampling sites. The table reports the mud cultivation technique (T—traditional tanks, S—mechanized silos, H—hybrid system); thermal mud (TM) distribution method (D—direct collection from tank, B—bagnomaria bucket, A—automated dispenser). Temperatures recorded in each sampling point (M—maturing mud tank, P—peloid, R—regenerating used mud) are also reported for the spring (SS) and autumn sampling (AS). Average temperature for each mud type, minimum and maximum values and first (Q1), second (Q2) and third quartile (Q3) are also reported.

ID	Cultivation Plant Typology	Thermal Mud Distribution	SS Temperature (°C)			AS Temperature (°C)		
			M	P	R	M	P	R
1	T	D	32.8	55.1	43.5	24.0	59.0	42.0
2	T	D	44.5	40.1	47.0	55.0	66.0	48.0
3	H	A	42.3	37.4	47.3	50.0	30.0	21.0
4	H	A	49.4	39.0	30.1	48.0	25.0	24.0
5	T	B	23.0	54.5	45.7	20.0	59.0	20.0
6	T	B	48.7	58.7	41.0	43.0	58.0	35.0
7	T	B	52.4	33.0	51.1	52.0	64.0	42.0
8	T	B	36.7	56.5	44.8	42.0	58.0	46.0
9	T	D	32.3	56.3	50.3	36.0	51.0	43.0
10	T	D	47.3	56.8	50.4	44.0	52.0	51.0
11	T	B	47.4	48.9	43.6	59.0	62.0	53.0
12	T	D	41.0	56.5	49.1	50.0	61.0	40.0
13	T	D	40.6	59.0	60.2	45.0	48.0	46.0
14	T	B	58.6	60.1	43.8	45.0	62.0	49.0
15	T	D	40.5	57.6	31.5	34.0	58.0	57.0
16	T	B	69.2	67.3	55.0	52.0	63.0	62.0
17	T	B	57.3	58.6	45.0	33.0	59.0	50.0
18	T	B	41.2	56.7	58.4	54.0	57.0	52.0
19	T	B	36.2	47.8	40.1	42.0	57.0	43.0
20	T	B	51.1	62.0	60.6	45.0	56.0	49.0
21	T	B	58.5	56.5	56.3	42.0	70.0	45.0
22	T	B	48.0	66.9	56.4	46.0	60.0	45.0
23	T	D	42.9	59.6	64.8	39.0	57.0	28.0
24	T	B	56.7	62.6	45.5	42.0	64.0	54.0
25	T	D	52.7	49.3	42.6	35.0	40.0	45.0
26	S	A	72.0	27.0	27.0	66.0	54.0	60.0
27	T	D	49.1	71.2	86.6	31.0	70.0	43.0
28	T	D	38.0	55.0	45.0	55.0	58.0	41.0
29	S	A	45.0	50.0	52.0	45.0	56.0	41.0
30	T	B	61.0	65.0	59.0	50.0	61.0	38.0
		average	47.2	54.2	49.1	44.1	56.5	43.8
		min	23.0	27.0	27.0	20.0	25.0	20.0
		max	72.0	71.2	86.6	66.0	70.0	62.0
		Q1	40.7	49.5	43.7	39.8	56.0	41.0
		Q2	47.4	56.5	47.2	45.0	58.0	45.0
		Q3	52.6	59.5	56.0	50.0	61.8	49.8

**Table 3:** Total viable counts (TVCs). Microbial counts at 22, 37 and 55 °C are reported for spring sampling (SS) and autumn sampling (AS) for all three mud typologies (M—maturing mud, P—peloid, R—regenerating used mud). Due to the high variability registered among different samples (25–78,400 colony forming units (CFU)/g), TVC values are conveniently divided into three classes, each reprised by a white-to-grey gradient. Graphical ranges are: <10^3^ CFU/g (22.6% of samples), 10^3^–10^4^ CFU/g (59.4% of samples) and >10^4^ CFU/g (18.0% of samples). Average CFU count, its minimum and maximum values and first (Q1), second (Q2) and third quartile (Q3) are also given.

ID	Spring Sampling (CFU/g)									Autumn Sampling (CFU/g)								
	TVC 22 °C			TVC 37 °C			TVC 55 °C			TVC 22 °C			TVC 37 °C			TVC 55 °C		
	M	P	R	M	P	R	M	P	R	M	P	R	M	P	R	M	P	R
**1**	10,750	3150	2800	3950	3750	4550	550	1950	4050	8450	4900	7750	2340	3000	7800	29,200	21,000	28,000
**2**	910	315	1845	855	420	1900	45	700	500	930	770	680	2050	1450	870	10,350	11,300	10,700
**3**	900	8200	5050	655	11,950	6700	300	11,100	6650	90	15,400	34,200	730	18,800	41,600	70	57,600	35,200
**4**	7350	6950	9900	11,350	8450	14,150	7250	6150	8350	24,000	27,400	17,000	15,200	8100	8300	12,700	8600	8850
**5**	4400	2500	3100	4300	3250	5700	440	2650	5400	3500	7500	5700	7400	9300	7100	12,650	78,200	21,750
**6**	6800	1805	1370	8050	1350	1425	17,300	745	5450	3750	3800	3100	3100	3000	2600	19,300	14,200	16,800
**7**	3450	7100	1390	3850	5600	800	4700	5800	4500	25	6500	11,200	40	3800	680	185	16,500	13,300
**8**	2490	2950	2385	2700	3450	1750	6250	3450	1590	8050	16,800	5800	6200	17,400	5450	21,100	32,400	17,200
**9**	5200	2090	1880	5350	1850	2350	10,650	5750	8250	1020	645	1710	4850	900	4100	54,600	4700	15,600
**10**	1765	65	1370	3400	370	2700	4800	330	3300	635	830	1090	1000	1070	3050	16,400	12,100	24600
**11**	1005	1850	665	905	1195	985	5800	560	755	1480	3400	2200	2500	9000	3550	2850	14,400	1700
**12**	235	985	740	770	1350	810	1105	1145	535	940	785	1460	1090	1400	2600	10,650	9400	46,800
**13**	270	525	515	465	495	555	265	570	515	1150	575	715	800	435	470	2350	3300	2700
**14**	2800	2950	3350	3550	6150	7400	3150	4750	4800	2050	2900	13,500	2600	2650	16,600	4600	6300	11,650
**15**	5400	5400	11,350	5050	6550	6350	7300	11,550	13,750	5300	2750	51,200	7750	5550	4450	78,400	51,200	43,200
**16**	4150	340	465	2700	370	445	2250	240	340	2800	2350	1450	1900	1150	1650	2050	285	300
**17**	3300	760	1630	3300	520	1165	4000	4250	1050	3300	1400	3050	2350	1020	6000	5500	19,000	55,200
**18**	585	630	205	585	635	270	732,5	315	460	1370	2300	3750	900	3300	6400	2950	3400	3110
**19**	2760	2610	2790	3100	3250	3750	4000	2400	3200	6400	2750	2750	4700	3500	4050	14,400	14,200	12,400
**20**	34,600	2250	4750	4850	2420	6800	22,600	2700	8800	5300	2450	3000	730	320	285	8500	10,000	2800
**21**	4950	4950	3600	5700	3500	3150	4450	4250	2550	4300	2900	1650	3150	3650	880	8550	8150	4550
**22**	715	190	1080	775	330	1110	400	275	445	715	1630	1700	1400	1950	3300	4700	5750	4100
**23**	8600	17,450	15,550	17,550	51,800	38,750	15,850	38,300	30,500	5150	9900	8800	7900	17,200	13,600	16,200	24,900	23,000
**24**	380	1165	1280	205	2250	1050	275	10,800	1800	785	510	400	1350	1500	4200	5950	2200	4050
**25**	1470	2050	8050	850	180	1035	850	2150	2450	665	810	1110	1050	11,800	2150	530	8300	3300
**26**	420	1065	2060	380	620	1705	2200	2150	1450	375	14,220	1575	860	9840	1580	7500	8400	5500
**27**	2550	1740	1940	530	195	250	12,150	2000	2500	8300	2200	12,100	635	135	195	4650	1220	4350
**28**	4810	4040	6240	470	610	640	12,400	8000	13,300	4600	6000	7550	470	575	410	21,450	20,550	25,400
**29**	34,000	12,400	26,400	5500	13,700	9300	3950	3400	5250	26,550	25,000	57,600	13,600	15,300	32,000	4800	5450	5800
**30**	3800	3400	11,900	4300	6300	8700	1150	1350	3350	2060	7000	8700	930	4700	6100	535	5250	4950
**average**	5361	3396	4522	3533	4762	4542	5239	4659	4861	4468	5879	9083	3319	5393	6401	12,789	15,942	15,229
**min**	235	65	205	205	180	250	45	240	340	25	510	400	40	135	195	70	285	300
**max**	34,600	17,450	26,400	17,550	51,800	38,750	22,600	38,300	30,500	26,550	27,400	57,600	15,200	18,800	41,600	78,400	78,200	55,200
**Q1**	934	1005	1370	771	543	998	762	845	1150	933	1458	1594	908	1213	1598	3363	5525	4163
**Q2**	3050	2170	2223	3200	2050	1825	3975	2525	3250	2430	2825	3075	1975	3150	3800	8000	9700	11,175
**Q3**	5138	3880	4975	4713	5138	6188	7000	5500	5438	5263	6875	8775	4313	8775	6325	15,750	18,375	22,688

**Table 4:** Total coliforms and *E. coli*. Microbial counts are reported for both indicator parameters. The first number refers to total coliforms whilst the one in round brackets to *E. coli* colonies. M—maturing mud, P—peloid, R—regenerating used mud.

Total Coliforms and *Escherichia coli* (CFU/g)						
	Spring Sampling			Autumn Sampling		
ID	M	P	R	M	P	R
1	1120 (85)	5	25 (20)	68	<1	191
2	<1	<1	<1	<1	<1	<1
3	<1	10	<1	<1	62	50
4	28	1	17	21	176	20
5	73	1	5	16 (2)	18	312
6	<1	<1	<1	<1	<1	<1
7	2	26	<1	<1	<1	8
8	<1	<1	<1	5	<1	6
9	66	<1	<1	<1	<1	<1
10	<1	<1	<1	<1	<1	<1
11	<1	<1	<1	<1	2	<1
12	<1	<1	<1	<1	2	<1
13	<1	<1	<1	<1	<1	<1
14	<1	2	<1	70	<1	48
15	2	<1	2 (24)	16	2	26
16	<1	<1	<1	<1	<1	<1
17	<1	<1	<1	<1	<1	<1
18	<1	<1	<1	<1	<1	<1
19	<1	4	<1	10	<1	2
20	<1	<1	<1	<1	<1	<1
21	<1	<1	<1	88	<1	26
22	<1	<1	<1	<1	<1	<1
23	<1	<1	<1	96	2	<1
24	<1	<1	<1	<1	<1	<1
25	<1	<1	<1	<1	<1	<1
26	17	1471	1020	4	<1	14
27	<1	<1	<1	100	<1	4
28	6	<1	<1	<1	<1	6
29	2	12	<1	14	<1	22
30	<1	<1	<1	<1	<1	<1

**Table 5:** Enterococci. Microbial counts are reported for the enterococci indicator parameter. M—maturing mud, P—peloid, R—regenerating used mud.

	Enterococci (CFU/g)					
	Spring Sampling			Autumn Sampling		
ID	M	P	R	M	P	R
1	145	15	20	177	206	269
2	<1	<1	<1	30	90	<1
3	<1	5	<1	<1	27	170
4	16	13	52	90	94	60
5	<1	<1	25	2	6	<1
6	41	14	18	8	2	8
7	208	24	288	<1	72	92
8	16	30	18	22	32	10
9	20	22	30	56	10	12
10	2	2	10	8	<1	4
11	2	66	36	14	<1	<1
12	<1	<1	<1	14	6	10
13	<1	<1	<1	2	2	<1
14	2	4	<1	<1	4	<1
15	16	8	14	8	6	16
16	<1	<1	<1	2	<1	2
17	14	<1	2	10	4	38
18	<1	<1	<1	2	4	<1
19	14	17	32	12	18	32
20	40	34	8	<1	<1	<1
21	6	16	14	26	6	4
22	10	8	4	2	<1	8
23	14	48	42	4	<1	24
24	40	20	126	22	38	26
25	1	7	9	<1	14	2
26	69	44	62	26	28	12
27	12	2	35	40	<1	6
28	106	56	72	38	18	86
29	<1	8	<1	<1	<1	28
30	<1	4	<1	<1	<1	4

**Table 6:** Sulfite-reducing clostridia. Microbial counts are reported. Similarly to the partition done for TVCs, 53.9% of clostridia counts are in the <100 CFU/g class (white background), 40.6% in the 100–1000 CFU/g (light grey) and 5.6% in the >1000 CFU/g one (dark grey). M—maturing mud, P—peloid, R—regenerating used mud.

	Sulfite-Reducing Clostridia (CFU/g)					
	Spring Sampling			Autumn Sampling		
ID	M	P	R	M	P	R
1	945	127	1635	300	395	590
2	135	30	170	54	34	28
3	55	395	1095	<1	160	740
4	1780	1535	2070	520	285	140
5	290	48	300	680	115	1525
6	480	95	240	300	15	35
7	520	350	112	<1	10	40
8	130	42	58	100	75	45
9	20	85	20	1220	120	170
10	35	10	110	44	4	24
11	10	40	16	<1	<1	13
12	44	220	160	22	16	120
13	25	20	35	20	120	20
14	<1	90	20	55	115	245
15	685	1045	335	450	225	65
16	350	25	5	85	<1	10
17	250	35	80	<1	20	25
18	35	10	5	<1	40	60
19	295	935	560	25	20	60
20	510	120	1380	290	120	<1
21	175	290	380	120	110	60
22	80	<1	95	60	17	24
23	860	185	345	40	690	230
24	20	125	145	165	210	270
25	15	80	340	<1	30	5
26	<1	140	1095	<1	360	320
27	14	3	10	95	7	18
28	114	99	52	72	14	32
29	595	240	920	95	180	210
30	85	95	160	<1	15	75

**Table 7:** Positive samples for *Pseudomonas aeruginosa* or dermatophytes. Dermatophytes were found in regenerating mud samples only. AS—autumn sampling; SS—spring sampling; M—maturing mud, P—peloid, R—regenerating used mud; CFU—colony forming units.

	ID	CFU/g
*P. aeruginosa*	AS-4P	40
	AS-9M	100
Dermatophytes	SS-1R	5
	SS-3R	5
	SS-4R	2
	SS-18R	2
	SS-26R	10

**Table 8:** Comparison of TVCs of mud samples between SS and AS. The table provides *p*-values and W statistics for the Wilcoxon signed rank test. Significant *p*-values (<0.05) are marked with asterisk (*). M—maturing mud, P—peloid, R—regenerating used mud.

		*p*-Value	W Stat
	22	0.158	141.0
M	37	0.905	212.0
	55	0.002 *	83.0
	22	0.020 *	119.0
P	37	0.225	173.5
	55	0.000 *	43.0
	22	0.026 *	125.0
R	37	0.102	153.0
	55	<0.001 *	32.0

**Table 9:** Correlation between thermal water temperature and TVCs of AS mud samples. The table provides *p*-values and ρ coefficients for Spearman’s R test. Significant *p*-values (<0.05) are marked with asterisk (*). AS—autumn sampling; M—maturing mud, P—peloid, R—regenerating used mud.

Sampling	Mud Type	Growth Temp. (°C)	*p*-Value	ρ
		22	0.027 *	−0.404
AS	M	37	0.025 *	−0.408
		55	0.037 *	−0.383
		22	0.333	−0.183
AS	P	37	0.209	−0.236
		55	0.441	−0.146
		22	0.010 *	−0.464
AS	R	37	0.121	−0.289
		55	0.028 *	−0.402

**Table 10:** Mann–Whitney U test for indicator parameter presence/absence in mud samples. *p*-values and U statistics are reported for the Mann–Whitney U test. All *p*-values are significant (<0.05) and thus are marked with asterisk (*). M—maturing mud, P—peloid, R—regenerating used mud.

Mud Type	Indicator Parameter	*p*-Value	U
**M**	Total coliforms	0.006 *	233.0
	*E. coli*	0.026 *	4
**U**	Total coliforms	0.005 *	182.5
**R**	Total coliforms	0.001 *	184.0
	Enterococci	0.022 *	214.5

**Table 11:** Presence/absence of indicator parameters and average thermal water temperature. Average temperature (AT) for positive and negative samples is reported. Presence of indicator parameters (>0 CFU/g) always occurred in samples with an average lower thermal water temperature. M—maturing mud, P—peloid, R—regenerating used mud.

Mud Type	Indicator Parameter	AT (°C) Presence	AT (°C) Absence
**M**	total coliforms	41.1	48.1
	*E. coli*	39.6	46.0
**P**	total coliforms	47.2	58.3
**R**	total coliforms	39.6	49.6
	Enterococci	45.4	49.3
